# Tracking longitudinal brain atrophy in dementia and cognitive impairment: insights from the Framingham Heart Study

**DOI:** 10.1002/alz70855_107409

**Published:** 2025-12-24

**Authors:** Habbiburr Rehman, Qiushan Tao, Ting Fang Alvin Ang, Jesse Mez, Rhoda Au, Lindsay A. Farrer, Douglas N Greve, Xiaoling Zhang, Wei Qiao Qiu

**Affiliations:** ^1^ Biomedical Genetics, Department of Medicine, Boston University Medical School, Boston, MA, USA; ^2^ Department of Pharmacology & Experimental Therapeutics, Boston University Chobanian & Avedisian School of Medicine, Boston, MA, USA; ^3^ Department of Neurology and Medicine, Boston University Chobanian & Avedisian School of Medicine, Boston, MA, USA; ^4^ Department of Anatomy & Neurobiology, Boston University Chobanian & Avedisian School of Medicine, Boston, MA, USA; ^5^ Framingham Heart Study, Framingham, MA, USA; ^6^ Department of Neurology, Boston University Chobanian & Avedisian School of Medicin, Boston, MA, USA; ^7^ Alzheimer's Disease Research Center, Boston University Chobanian & Avedisian School of Medicine, Boston, MA, USA, Boston, MA, USA; ^8^ Framingham Heart Study, Boston University School of Medicine, Boston, MA, USA; ^9^ Department of Epidemiology, Boston University School of Public Health, Boston, MA, USA; ^10^ Department of Neurology, Boston University Chobanian & Avedisian School of Medicine, Boston, MA, USA; ^11^ Department of Neurology and Ophthalmology, Boston University Chobanian & Avedisian School of Medicine, Boston, MA, USA; ^12^ Department of Biostatistics, Boston University School of Public Health, Boston, MA, USA; ^13^ Athinoula A. Martinos Center for Biomedical Imaging, Massachusetts General Hospital, Harvard Medical School, Charlestown, MA, USA; ^14^ Department of Psychiatry, Boston University Chobanian & Avedisian School of Medicine, Boston, MA, USA; ^15^ Alzheimer's Disease Research Center, Boston University Chobanian & Avedisian School of Medicine, Boston, MA, USA

## Abstract

**Background:**

Dementia due to Alzheimer's disease (AD) is a degenerative neurological disorder that results in abnormal protein degradation and loss of function in the brain. However, the association of longitudinal changes in brain atrophy with dementia and cognitive impairment is unclear.

**Method:**

We included Framingham Heart Study (FHS) Generation 01 and 02 participants with available FreeSurfer MRI (1126 features) data with at least two visits (*n* = 756, cognitive normal (CN)=533, mild cognitive impairment (MCI) =102, and Dementia=121). First, linear mixed effect model was used to identify the MRI features associated (FDR<0.05) with MCI and dementia. Second, the Assorted Linear functions for ANOVA Simultaneous Component Analysis (ALASCA) method was applied to generate the longitudinal principal components (PCs).

**Result:**

We identified 52 and 143 features associated with MCI and dementia, respectively. First three PCs generated from MCI MRI features (M‐PCs) which accounting M‐PC1=73.16%, M‐PC2=16.63%, and M‐PC3=6.62% of variations are significant and first two PCs generated from 143 dementia features (D‐PCs) are significant which accounting D‐PC1=86.55% and D‐PC2=6.19% of the variation in the data. M‐PC1 and D‐PC1 decrease at each MRI scan in both MCI and dementia patients compared to CN individuals. However, M‐PC2 and D‐PC2 are increasing in MCI participants and decreasing in dementia patients. Both M‐PC1 and D‐PC1 also significantly associated with plasma biomarkers (*p*‐tau181 [M‐PC1: β=‐1.46, *p* = 0.0007 and D‐PC1: β=‐2.21, *p* = 0.0026], t‐tau [M‐PC1: β=‐0.85, *p* = 0.0097 and D‐PC1: β=‐1.29, *p* = 0.025]) and cognitive function (language [M‐PC1: β=1.25, *p* = 1.7e‐05 and D‐PC1: β=1.31, *p* = 6.2e‐07], memory [M‐PC1: β=0.92, *p* = 0.004 and D‐PC1: β=1.71, *p* = 3.6e‐09], executive function [M‐PC1: β=0.32, *p* = 0.27 and D‐PC1: β=0.75, *p* = 0.004], MMSE [M‐PC1: β=0.39, *p* = 0.017 and D‐PC1: β=0.6, *p* = 0.03]). Highly correlated MRI features with M‐PC1 include different brain regions “pulvinar”, “subiculum”, “amygdala”, “hippocampus”, etc. and D‐PC1 includes “entorhinal”, “hippocampus”, “pulvinar”, “amygdala”, etc. All results were adjusted for age at MRI, sex, education, and APOE ɛ4 risk factors.

**Conclusion:**

This study identified atrophy developing MRI features (52 MCI and 143 dementia) from different brain regions with clinical diagnosis, cognitive decline, and plasma AD biomarkers. Our findings suggest that these brain regions will be important in better understanding dementia heterogeneity, particularly as clinical phases or distinct biological subtypes.

